# Identification of plasma lipid metabolism and potential biomarkers in patients with different coronary occlusive acute myocardial infarction

**DOI:** 10.3389/fcell.2025.1575431

**Published:** 2025-08-14

**Authors:** Hao Chen, Qingyi Zhang, Ai Zhou, Li Zhang, Xing Li

**Affiliations:** ^1^ Department of Cardiology, Wuxi No. 2 People’s Hospital of Jiangnan University Medical Center, Wuxi, Jiangsu, China; ^2^ Department of Cardiology, Affiliated Changzhou Children’s Hospital of Nantong University, Changzhou, China; ^3^ Hongqiao International Institute of Medicine, Tongren Hospital, Shanghai Jiao Tong University School of Medicine, Shanghai, China

**Keywords:** myocardial infarction, lipid molecules, lipidomic, phosphatidylcholine, lysophosphatidylcholines

## Abstract

Acute myocardial infarction (AMI) is the main cause of death worldwide. We aim to compare the differences in plasma lipid metabolites between AMI patients and normal controls to search for biomarker molecules for AMI with different infarct sites. We enrolled 12 patients in Group A (left coronary artery occlusion), 15 in Group B (right coronary artery occlusion), and 14 in Group C (normal controls) from June 2020 to June 2021. Non-targeted lipidomic analysis was performed and a total of 93 differential lipid molecules were identified. Diagnostic efficiency was evaluated by receiver operating curve. Compared with Group C, there were nine lipid molecules with AUC>0.8 in Groups A and B. Compared with Group B, Group A had six lipid molecules with AUC>0.8. These lipid molecules belonged to the LPC, PC, TG, and DG classes. We focused on LPC (20:4) as a biomarker in AMI.

## 1 Introduction

Cardiovascular disease and its associated complications are the leading cause of death and disability worldwide, causing 17.8 million deaths each year (GBD, 2017 Causes of Death Collaborators, 2018). At present, the prevalence of cardiovascular disease in China is on the rise. It is estimated that there are 330 million cardiovascular patients, of whom coronary heart disease accounts for 11 million (Sanz and Fayad, 2008). Acute myocardial infarction (AMI) is one of the most serious and harmful types of coronary heart disease. Its pathogenesis is the formation of atherosclerotic plaques in the coronary arteries driven by lipoprotein deposition, which is involved in inflammatory responses, immune responses, and other factors. Vulnerable plaques rupture or erode, triggering a coagulation cascade which leads to acute thrombosis, thereby blocking coronary blood flow and causing acute ischemic damage or even necrosis of a large area of myocardial cells (Sanz and Fayad, 2008). Although medical technology has made great progress, AMI is still a killer that threatens human health. In recent decades, more and more metabolomic techniques have been used to demonstrate the metabolic changes in diseases such as atherosclerosis (Wang et al., 2017), myocardial ischemia (Al-Rasadi et al., 2018; Lanfear et al., 2017), heart failure (Lanfear et al., 2017), and hypertension (Zheng et al., 2013). These new metabolomic techniques provide novel approaches to exploring the pathogenesis of cardiovascular disease and to search for biologically active substances. Lipidomics, a branch of metabolomics, is the systems-based study of all lipids, their interacting molecules, and their functions in cells (Sud et al., 2007). As dyslipidemia is a vital risk factor for AMI, we chose lipidomic analysis to further explore changes of lipid metabolism in AMI.

In our daily clinical work, we found that the clinical presentation and long-term prognosis of patients with left or right coronary artery occlusion are quite different. American Cardiovascular Association guidelines for myocardial infarction indicate that anterior wall myocardial infarction is an important indicator for the prognosis risk assessment of MI patients (Arnett et al., 2019). The vessel most commonly involved in all MI patients is the left anterior descending (LAD) artery, and thus the anterior wall supplied by the LAD is also the most common in myocardial infarction. The anterior descending artery accounts for two-thirds of the blood supply to the entire heart. When myocardial infarction occurs in the anterior wall, its severity is often greater than that of non-anterior myocardial infarction, and the prognosis is worse. Adverse left ventricular remodeling and long-term major cardiovascular events risk is higher. However, the factors affecting cardiac structure, function and prognosis after myocardial infarction also include infarct volume and transmural degree. After excluding these factors that affect myocardial infarction, it is still worth exploring whether the location of infarction has an independent impact on myocardial infarction. Most previous studies on myocardial infarction have been divided into a myocardial infarction patient group and a normal control group. Considering the specificity of anterior wall infarcts mentioned above, there were three groups of subjects in our study: Group A, anterior wall infarction caused by the anterior descending artery; Group B, inferior wall infarction caused by the right coronary artery; Group C, normal control group.

Our study applied an untargeted lipidomic profiling strategy based on ultraperformance liquid chromatography coupled with mass spectrometry (UPLC-MS) to identify lipidomic features of the arterial plasma samples obtained from AMI patients with anterior wall infarction, inferior wall infarction, and healthy controls to observe the changes in lipid metabolism in those with a different infarct region and to identify biomarker molecules that may affect the development process of myocardial infarction.

## 2 Materials and methods

### 2.1 Study subjects

We included patients who underwent coronary angiography with AMI diagnosis from 30 January 2020 to 30 June 2021 in the Department of Cardiology of Wuxi No. 2 People’s Hospital of Nanjing Medical University due to chest pain, ischemic electrocardiogram (ECG) changes, and elevated myocardial markers. The diagnosis of AMI is based on the globally harmonized definition of myocardial infarction published by the European Society of Cardiology in 2018 (Arnett et al., 2019)—a typical elevation and/or decrease in biomarkers of myocardial necrosis, at least once exceeding the 99th percentile of healthy people, and evidence of at least one of the following: (1) clinical symptoms of myocardial ischemia; (2) new myocardial ischemia changes (ST-segment elevation or decrease) on ECG; (3) pathological Q on ECG waves; (4) imaging evidence of new loss of myocardial viability or regional wall motion abnormalities. Confirmed by percutaneous coronary angiography, 12 patients with left coronary artery occlusion and 17 with right coronary artery occlusion were enrolled. The control group underwent coronary angiography in the same period and showed normal coronary arteries—a total of 15 people. After quality control and PCA, two outliers in Group B and one in Group C were excluded (Supplementary Figure S1), so 41 samples were finally included for analysis. The inclusion criteria were (1) AMI discovered for the first time and did not receive reperfusion therapy; (2) family members and patients agreed to be enrolled. The exclusion criteria were: previous myocardial infarction; heart failure; history of malignant tumor; chronic obstructive pulmonary disease; arrhythmia high atrioventricular block, ventricular fibrillation; congenital heart disease, cardiomyopathy, rheumatic heart disease; abnormal renal function; blood system diseases; abnormal thyroid function; pregnant or lactating women; people with bacterial, viral infection, acute and chronic inflammation, and immune diseases.

Written informed consent was obtained from each subject before their participation in the study. Personal information was omitted, and identification was replaced by their health examination number. The research protocol was approved by the ethics committee of the Wuxi No. 2 People’s Hospital of Nanjing Medical University (2021-Y-9).

### 2.2 Diagnosis of the culprit vessel of AMI

**FIGURE 1:**
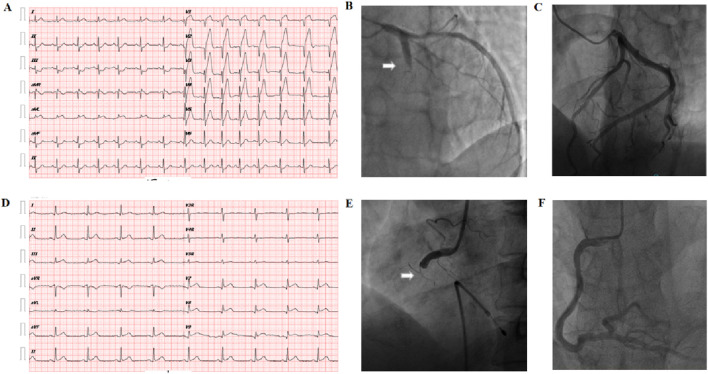
Diagnosis of the culprit vessel of AMI. **(A)** Abnormal Q waves in the V1–V4 chest leads and extensive ST-segment elevation in the anterior leads on the body surface electrocardiogram suggest acute anterior myocardial infarction. **(B)** Arrow: coronary angiography shows left acute occlusion of the proximal segment of the anterior descending coronary artery. **(C)** Left coronary artery of healthy controls: no obvious atherosclerotic plaque formation was found in the anterior descending and circumflex branches. **(D)** Electrocardiogram on the body surface showed Ⅱ, Ⅲ, avF leads combined ST-segment elevation, suggesting acute inferior wall myocardial infarction. **(E)** Arrow: acute occlusion of the proximal right coronary artery; **(F)** right coronary artery of healthy control: no obvious atherosclerotic plaque formation in the whole right coronary artery.

Emergency coronary angiography (CAG) and percutaneous coronary intervention (PCI) were performed in the cardiac catheterization laboratory through the radial artery by four experienced coronary interventionists. Enteric-coated aspirin 300 mg and ticagrelor 180 mg were taken before the operation, and the patients were sent directly from the emergency room to the catheterization room. The right radial artery was routinely punctured with the Seldinger method placed into the 6F arterial sheath. The infarction-related vessels were determined by multi-projection angle selective coronary angiography using the Judkins method with a cardiovascular imaging machine. Judgment criteria for infarcted relative artery (IRA) were: (1) visible thrombus shadow; (2) visible ulcers formed by plaque detachment; (3) judgment of “culprit blood vessel”—ST-segment changes in the corresponding leads of the ECG and abnormal segment motion of the left ventricular wall. Patients with anterior or anterior septal myocardial infarction due to left coronary artery occlusion were defined as “Group A.” Patients with inferior wall or right ventricular myocardial infarction due to right coronary artery occlusion were defined as “Group B.” Healthy controls were defined as three coronary vascular stenosis <50% as “Group C.” The typical ECG and angiographic findings of anterior wall infarction and inferior wall infarction are shown in Figure 1.

### 2.3 Collection of blood samples and clinical data

After successful radial artery puncture, a 5-mL sample of artery blood was immediately placed in a heparin anticoagulant tube, left to stand at room temperature for 30 min, centrifuged at 1200 *g* for 10 min, and 300 uL of the supernatant was kept and frozen at −80°C thereafter.

**FIGURE 2:**
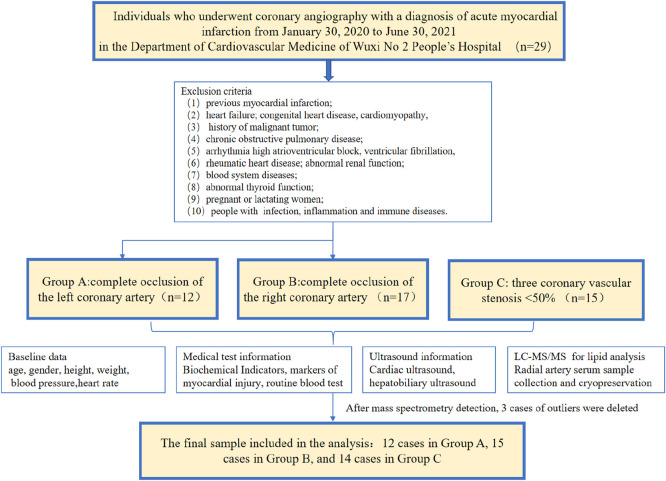
Flowchart of study design.

Baseline data such as age, gender, height, weight, blood pressure, renal function, liver function, electrolytes, markers of myocardial injury, routine blood test, and cardiac ultrasound examination results were collected for all subjects. The flow chart is shown in Figure 2. The information regarding the collection of clinical samples is as follows. 1) Sample collection: all samples were collected within 12 h of the onset of acute chest pain caused by myocardial ischemia. 2) Medication administration: blood was drawn before coronary angiography, with all patients receiving a loading dose of 300 mg aspirin, 180 mg ticagrelor, and 20 mg rosuvastatin. 3) Reperfusion therapy: all patients underwent emergency coronary angiography, with percutaneous coronary intervention (PCI) performed when necessary.

### 2.4 Preparation of quality control samples

Equal amounts of samples from each group were mixed as quality control samples, which were used to determine the state of the instrument before injection and to balance the chromatography mass spectrometry system. After every eight real samples were run, a quality control sample was inserted to evaluate the stability of the system during the entire experiment.

### 2.5 Sample preparation and lipid extraction

Lipids were extracted according to the MTBE method, where samples were first spiked with an appropriate amount of internal lipid standards and then homogenized with 200 µL water and 240 µL methanol. After that, 800 µL of MTBE was added and the mixture was subjected to 20 min ultrasound at 4°C, followed by sitting still for 30 min at room temperature. The solution was centrifuged at 14000 *g* for 15 min at 10°C, and the upper organic solvent layer was obtained and dried under nitrogen.

### 2.6 LC-MS/MS method for lipid analysis

Reverse-phase chromatography was selected for LC separation using a CSH C18 column (1.7 µm, 2.1 mm × 100 mm, Waters). The lipid extracts were re-dissolved in 200 µL 90% isopropanol/acetonitrile, centrifuged at 14000 *g* for 15 min, and 3 µL of the sample was then injected. Solvent A was acetonitrile–water (6:4, v/v) with 0.1% formic acid and 0.1 Mm ammonium formate, and solvent B was acetonitrile–isopropanol (1:9, v/v) with 0.1% formic acid and 0.1 Mm ammonium formate. The initial mobile phase was 30% solvent B at a flow rate of 300 μL/min. It was held for 2 min and then linearly increased to 100% solvent B in 23 min, followed by equilibrating at 5% solvent B for 10 min.

Mass spectra were acquired by Q-Exactive Plus in positive and negative mode. ESI parameters were optimized and preset for all measurements as follows: source temperature, 300°C; capillary temp, 350°C; ion spray voltage was set at 3000V; S-Lens RF level was set at 50%; scan range of the instruments was set at m/z 200–1800.

### 2.7 Data preprocessing and annotation

The data were normally distributed and homoskedastic. One-way analysis of variance (one-way ANOVA) was used to identify the significantly changed (p < 0.05) lipid metabolites among the three groups. If a significant difference was observed, we used the Bonferroni *post hoc* test to check which two groups differed significantly. A principal component analysis (PCA) model was used to observe the overall distribution trend and degree of difference between the three groups, providing a comprehensive view of clustering trends and outliers, and then a partial least-squares discriminant analysis (partial least-squares) was used. The orthogonal partial least squares discriminant analysis (OPLS-DA) model was performed to visualize the discrimination between the different groups. Lipid metabolites with variable importance in projection (VIP) value >1 were viewed as the differential lipid metabolites. Markedly changed lipid metabolites identified by both one-way ANOVA and OPLS-DA model were viewed as the key differential lipid metabolites. Meanwhile, we used a hierarchical clustering algorithm to investigate whether these key differential lipid metabolites could correctly cluster the samples. A receiver operating characteristic (ROC) curve was used to evaluate the diagnostic efficiency of the selected differential lipid molecules. Finally, if one key lipid metabolite was also significantly changed between the three groups (P value ≤ 0.05, one-way ANOVA; VIP>1, OPLS-DA; AUC>0.8, ROC), then this could be ultimately selected as candidate biomarker. We use two-factor correlation analysis to judge the linear relationship between two variables. When the P value ≤ 0.05, it was considered statistically different. When the Pearson correlation was 0.4–0.6, the two factors were considered to have a moderate degree of correlation, 0.6–0.8 was considered strongly correlated, and 0.8–1.0 was considered very strongly correlated.

## 3 Results

### 3.1 Baseline characteristics of the study population

The clinical characteristics of the participants are shown in Table 1. The study population comprised 41 individuals: 12 in Group A (age 60.25 ± 2.84 years), 15 in Group B (age 63.94 ± 2.98 years), and 14 in Group C (age 55.29 ± 4.81 years). The comparison showed that there was no statistical difference in gender ratio, age, renal function, blood glucose level, electrolytes, diastolic blood pressure, and other indicators among the three groups of subjects, while systolic blood pressure, blood lipid metabolism-related APOA, myocardial injury-related indicators, left ventricular ejection fraction, and inflammation-related indicators (leukocytes, neutrophils, lymphocytes, neutrophils/lymphocytes, and high-sensitivity C-reactive protein) were statistically different (P > 0.05).

**TABLE 1:** Clinical characteristics of the participants in the three groups.

General information	Group A (n = 12)	Group B (n = 15)	Group C (n = 14)	P value
Age(y)	60.25 ± 2.84 (44–73)	63.94 ± 2.98 (33–86)	55.29 ± 4.81 (32–84)	0.237
Sex (Male%)	10 (83.3%)	12 (70.5%)	11 (73.3%)	0.725
SBP (mmHg)	123.17 ± 4.57^a^ (113.09–133.24)	125.71 ± 5.23^b^ (113.24–137.02)	142.27 ± 5.14 (129.72–148.42)	0.025*
DBP (mmHg)	75.67 ± 3.82 (67.24–84.09)	75.53 ± 3.23 (67.51–82.22)	80.93 ± 3.91 (71.53–85.19)	0.490
Cr (umol/L)	64.31 ± 4.31 (54.82–73.80)	64.78 ± 5.64 (52.55–79.56)	63.22 ± 4.60 (58.81–71.02)	0.979
eGFR (ml/min/L)	99.16 ± 3.87 (90.63–107.69)	96.05 ± 4.82 (82.84–104.89)	101.11 ± 3.21 (96.26–108.42)	0.729
Na^+^(mmol/L)	139.61 ± 0.66^a^ (138.16–141.07)	138.51 ± 0.51^b^ (137.15–139.60)	141.29 ± 0.66 (139.74–142.88)	0.007*
K^+^(mmol/L)	3.81 ± 0.11 (3.58–4.05)	3.99 ± 0.12 (3.70–4.28)	3.92 ± 0.08 (3.73–4.13)	0.552
Lipid Metabolism				
Chol (mmol/L)	4.85 ± 0.54 (3.65–6.05)	4.47 ± 0.25 (3.87–5.03)	4.44 ± 0.24 (3.94–5.08)	0.677
TG (mmol/L)	1.59 ± 0.28 (0.96–2.22)	1.46 ± 0.22 (1.02–1.49)	1.85 ± 0.17 (1.40–2.23)	0.501
HDL (mmol/L)	1.24 ± 0.08 (1.06–1.42)	1.30 ± 0.05 (1.22–1.46)	1.29 ± 0.053 (1.20–1.43)	0.762
LDL (mmol/L)	3.34 ± 0.42 (2.40–4.27)	2.80 ± 0.20 (2.30–3.22)	2.76 ± 0.224 (2.30–3.34)	0.313
APOA (g/L)	1.07 ± 0.04^a^ ^,^ ^c^ (0.96–1.17)	1.20 ± 0.04 (1.10–1.33)	1.25 ± 0.03 (1.17–1.34)	0.036*
APOB(g/L)	1.13 ± 0.12 (0.85–1.41)	0.99 ± 0.07 (0.79–1.14)	0.94 ± 0.75 (0.75–1.15)	0.386
APOE (mg/L)	45.26 ± 6.16 (31.68–58.84)	42.74 ± 3.36 (35.45–44.58)	41.75 ± 3.89 (33.47–51.90)	0.861
Lpa (mg/L)	209.16 ± 46.34 (107.15–311.18)	341.29 ± 77.94 (160.76–535.64)	159.83 ± 31.03 (81.57–232.42)	0.105
Myocardial injury marker				
CK(U/L)	1,601.30 ± 569.22^a^ (348.43–2,854.16)	1,354.45 ± 240.76^b^ (767.25–1892.12)	136.53 ± 53.27 (5.60–278.77)	0.025*
CKMB(U/L)	134.81 ± 51.63^a^ (21.17–248.46)	92.49 ± 14.96^b^ (59.09–129.60)	11.47 ± 2.11 (5.69–15.13)	0.035*
cTnI (ng/mL)	23.32 ± 8.72^a^ (4.11–42.53)	26.18 ± 8.14^b^ (4.19–42.48)	<0.01 ± 0.00 (<0.01)	0.016*
cTnT (ng/L)	18474.45 ± 1,016.28^a^ ^,^ ^c^ (345.30–100000)	2,872.96 ± 757.51^b^ (907.99–4,151.51)	20.26 ± 3.10 (18.42–22.14)	0.048*
LVEF (%)	49.67 ± 2.93^a^ (43.21–56.13)	53.41 ± 1.68^b^ (48.92–56.95)	60.20 ± 0.20 (56.75–60.68)	0.001**
Inflammation				
GAl (g/L)	13.42 ± 1.59 (9.92–16.93)	13.42 ± 1.40 (9.70–15.97)	10.57 ± 0.60 (9.28–12.15)	0.305
GHb(%)	6.83 ± 0.61 (5.49–8.17)	6.81 ± 0.44 (5.63–7.53)	3.21 ± 0.43 (2.61–5.12)	0.848
hs-CRP (mg/L)	20.13 ± 8.84 (0.66–39.61)	16.43 ± 8.35 (4.78–12.09)	1.91 ± 0.62 (0.38–3.43)	0.241
WBC(*10^9^/L)	8.79 ± 0.87^a^ (6.87–10.71)	10.11 ± 0.64^b^ (8.42–11.48)	6.51 ± 0.46 (5.48–7.65)	0.001**
NE (*10^9^/L)	7.01 ± 0.92^a^ (4.99–9.04)	7.61 ± 0.66^b^ (5.87–9.01)	3.82 ± 0.30 (3.13–4.56)	0.000**
Lym (*10^9^/L)	1.34 ± 0.15 (1.00–1.69)	1.88 ± 0.25 (1.27–2.54)	2.10 ± 0.19 (1.68–2.58)	0.075*
N/L	6.50 ± 1.36^a^ (3.49–9.52)	5.94 ± 1.24^b^ (3.01–9.08)	1.99 ± 1.45 (1.40–2.57)	0.009*

Abbreviations: SBP, systolic blood pressure; DBP, diastolic blood pressure; Cr, creatinine; eGFR, estimated glomerular filtration rate; Chol, cholesterol; TG, triglycerides; HDL, high-density cholesterol; LDL, low-density cholesterol; CK, creatine kinase; CKMB, creatine kinase-MB; cTnI, troponin I; cTnT, troponin T; LVEF, left ventricular ejection fraction; GAl, glycated plasma album; GHb, glycated hemoglobin; hs-CRP, high sensitive C-reactive protein; WBC, white blood cell count; NE, neutrophil granulocyte; Lym, lymphocyte; N/L, neutrophil granulocyte/lymphocyte. *P<0.05, **P<0.01.

Intergroup comparisons among the three groups were performed using one-way ANOVA. A p-value < 0.05 in the table indicates statistically significant differences among at least two groups.

Subsequent pairwise comparisons (*post hoc* tests) were conducted using the LSD method to identify specific group differences.

^a^ Denotes statistically significant differences between Groups A and C.

^b^ Indicates statistically significant differences between Groups B and C.

^c^ Represents statistically significant differences between Groups A and B.

### 3.2 Lipid composition analysis of lipid metabolites

The lipid profiles of the 12 participants with AMI due to left coronary artery occlusion, 15 participants with AMI due to right coronary artery occlusion, and the 14 participants with normal coronary arteries were analyzed. In total, 20 lipid classes and 1,355 signals were obtained after data processing, as described in “Methods” above. Differential lipid molecules were defined as the fold change (FC) > 1.5 or <0.67, P value < 0.05. The differentially expressed lipid molecules are shown in Table 2.

**TABLE 2:** Change of lipids between different groups.

Lipid ion	Ion formula	CalMz	RT-(min)	Fold change	P-value	VIP
Group A and Group C						
ChE (18:3)+NH4	C45 H78 O2 N1	664.603	19.673	0.516	0.033	3.113
DG (18:1_20:4)+NH4	C41 H74 O5 N1	660.556	12.059	0.474	0.046	1.271
DG (18:2_20:4)+NH4	C41 H72 O5 N1	658.541	11.297	0.548	0.039	1.278
DG (20:0_22:6)+NH4	C45 H80 O5 N1	714.603	17.383	2.678	0.001	1.989
DG (36:8)+H	C39 H61 O5	609.451	11.387	1.694	0.007	1.318
DG (42:6)+NH4	C45 H80 O5 N1	714.603	17.604	2.536	0.001	2.044
LPC (18:0)+HCOO	C27 H55 O9 N1 P1	568.362	3.413	0.625	0.013	2.303
LPC (18:1)+HCOO	C27 H53 O9 N1 P1	566.346	2.747	0.663	0.006	1.561
LPC (18:2)+H	C26 H51 O7 N1 P1	520.340	2.199	0.602	0.014	3.037
LPC (20:3)+H	C28 H53 O7 N1 P1	546.355	3.673	0.590	0.001	1.103
LPC (20:4)+HCOO	C29 H51 O9 N1 P1	588.331	2.131	0.478	0.000	1.865
LPC (20:5)+H	C28 H49 O7 N1 P1	542.324	2.205	0.625	0.012	1.009
LPC (22:6)+HCOO	C31 H51 O9 N1 P1	612.331	2.028	0.474	0.001	1.105
LPE (18:2)-H	C23 H43 O7 N1 P1	476.278	2.300	0.623	0.045	1.069
LPE (20:4)-H	C25 H43 O7 N1 P1	500.278	2.215	0.570	0.012	1.030
PC (10:0e_8:0)+H	C26 H55 O7 N1 P1	524.371	3.685	0.506	0.004	2.642
PC (18:0_22:6)+HCOO	C49 H85 O10 N1 P1	878.592	10.804	0.637	0.016	2.154
PC(18:1_20:4)+HCOO	C47 H83 O10 N1 P1	852.576	10.177	0.650	0.011	2.430
PC (18:3_18:2)+HCOO	C45 H79 O10 N1 P1	824.545	8.757	0.502	0.008	2.189
PC (23:1_10:1)+Li	C41 H78 O8 N1 P1 Li1	750.562	11.174	1.635	0.000	2.158
PC (38:4)+H	C46 H85 O8 N1 P1	810.601	10.812	0.642	0.021	1.016
PC(8:0e_8:0)+H	C24 H51 O7 N1 P1	496.340	2.605	0.600	0.001	4.070
PC (8:1e_10:0)+H	C26 H53 O7 N1 P1	522.355	2.743	0.542	0.001	1.903
PC (8:1e_10:1)+HCOO	C27 H51 O9 N1 P1	564.331	2.203	0.551	0.005	5.284
PE (37:2)+Li	C42 H80 O8 N1 P1 Li1	764.578	11.494	1.806	0.001	4.945
PE (50:2)+Li	C55 H106 O8 N1 P1 Li1	946.781	18.639	0.408	0.033	1.675
PG (31:2e)+NH4	C37 H75 O9 N1 P1	708.517	11.487	1.957	0.001	7.577
PG (32:6)+NH4	C38 H67 O10 N1 P1	728.450	3.715	2.230	0.005	1.186
PI (16:0_20:4)-H	C45 H78 O13 N0 P1	857.519	8.951	0.620	0.030	2.031
PS (17:1_22:5)+Li	C45 H76 O10 N1 P1 Li1	828.536	8.727	2.725	0.000	1.362
PS (24:3)-H	C30 H51 O10 N1 P1	616.326	2.116	1.731	0.034	1.280
PS (34:0_18:2)+K	C58 H110 O10 N1 P1 K1	1,050.750	2.727	0.479	0.003	1.232
PS (39:3)+Na	C45 H82 O10 N1 P1 Na1	850.557	10.996	2.673	0.031	3.124
TG (16:0_16:1_18:2)+NH4	C53 H100 O6 N1	846.755	19.328	0.586	0.050	2.406
TG (16:1_18:2_18:3)+NH4	C55 H98 O6 N1	868.739	17.412	0.484	0.042	1.130
TG (18:1_18:1_20:3)+NH4	C59 H108 O6 N1	926.817	20.978	0.570	0.050	1.043
TG (18:1_18:1_20:5)+NH4	C59 H104 O6 N1	922.786	19.105	0.595	0.042	1.819
Group B and Group C						
DG (16:0_20:4)+NH4	C39 H72 O5 N1	634.541	11.981	0.559	0.028	1.349
DG (18:1_20:3)+NH4	C41 H76 O5 N1	662.572	12.509	0.617	0.045	1.508
DG (18:1_20:4)+NH4	C41 H74 O5 N1	660.556	12.059	0.546	0.034	2.332
DG (18:2_20:4)+NH4	C41 H72 O5 N1	658.541	11.297	0.550	0.009	2.771
DG (18:3e)+NH4	C21 H40 O4 N1	370.295	1.681	2.074	0.005	3.153
DG (20:3e)+NH4	C23 H44 O4 N1	398.326	2.053	1.890	0.020	1.896
DG (20:4e)+NH4	C23 H42 O4 N1	396.311	1.751	2.400	0.008	1.571
DG (24:3e)+NH4	C27 H52 O4 N1	454.389	3.676	2.032	0.003	1.034
DG (33:2e)+H	C36 H69 O4	565.519	21.917	0.407	0.009	1.037
DG (36:2e)+H	C39 H75 O4	607.566	22.972	0.469	0.036	1.230
DG (8:0e_10:4)+NH4	C21 H38 O4 N1	368.280	1.516	2.402	0.007	3.224
LPC (18:0)+HCOO	C27 H55 O9 N1 P1	568.361	3.412	0.637	0.046	3.638
LPC (20:4)+HCOO	C29 H51 O9 N1 P1	588.331	2.131	0.635	0.017	1.947
MG (18:2)+NH4	C21 H42 O4 N1	372.311	1.922	1.619	0.021	1.207
TG (14:0_18:2_18:2)+NH4	C53 H98 O6 N1	844.739	18.410	0.394	0.036	2.485
TG (15:0_16:0_18:1)+NH4	C52 H102 O6 N1	836.770	20.939	0.469	0.044	1.263
TG (15:0_18:1_18:2)+NH4	C54 H102 O6 N1	860.770	19.944	0.598	0.017	2.145
TG (15:0_18:2_18:2)+NH4	C54 H100 O6 N1	858.755	18.890	0.569	0.016	1.437
TG (16:0_16:1_18:2)+NH4	C53 H100 O6 N1	846.755	18.272	0.524	0.040	1.004
TG (16:0_17:0_18:1)+NH4	C54 H106 O6 N1	864.801	21.909	0.506	0.030	1.493
TG (16:0_17:1_18:1)+NH4	C54 H104 O6 N1	862.786	20.948	0.569	0.040	1.970
TG (16:0_18:1_18:3)+Na	C55 H98 O6 Na1	877.726	19.773	0.639	0.013	1.501
TG (16:0_18:1_22:6)+NH4	C59 H104 O6 N1	922.786	19.661	0.630	0.039	2.061
TG (16:0_18:2_18:3)+NH4	C55 H100 O6 N1	870.755	18.587	0.644	0.029	4.500
TG (16:1_18:2_18:3)+NH4	C55 H98 O6 N1	868.739	17.412	0.505	0.021	2.198
TG (17:0_18:1_18:1)+NH4	C56 H108 O6 N1	890.817	21.907	0.574	0.023	1.751
TG (18:0_16:0_18:0)+NH4	C55 H110 O6 N1	880.833	22.998	0.360	0.013	1.898
TG (18:1_17:1_18:2)+NH4	C56 H104 O6 N1	886.786	20.078	0.632	0.034	1.457
TG (18:1_18:1_20:4)+NH4	C59 H106 O6 N1	924.801	20.338	0.528	0.040	2.534
TG (18:1_18:1_20:5)+NH4	C59 H104 O6 N1	922.786	19.105	0.661	0.038	2.829
TG (18:1_18:2_18:3)+NH4	C57 H102 O6 N1	896.770	18.761	0.393	0.024	2.355
TG (18:1_18:2_22:1)+NH4	C61 H114 O6 N1	956.864	19.756	0.591	0.011	1.498
TG (18:1_18:2_22:2)+NH4	C61 H112 O6 N1	954.848	18.939	0.500	0.017	1.070
TG (18:1_18:2_22:2)+NH4	C61 H112 O6 N1	954.848	18.474	0.548	0.035	1.823
TG (18:2_18:2_20:4)+NH4	C59 H102 O6 N1	920.770	18.101	0.470	0.038	1.415
TG (18:2_18:2_22:6)+NH4	C61 H102 O6 N1	944.770	17.729	0.509	0.008	1.269
TG (18:3_18:2_18:2)+NH4	C57 H100 O6 N1	894.755	18.444	0.478	0.011	1.020
TG (18:3_18:2_18:2)+NH4	C57 H100 O6 N1	894.755	17.542	0.597	0.047	2.814
TG (18:3_18:2_20:3)+NH4	C59 H102 O6 N1	920.770	17.802	0.566	0.024	2.692
TG (22:2_18:2_18:2)+NH4	C61 H110 O6 N1	952.833	17.846	0.360	0.003	1.022
TG (22:4_18:2_18:2)+NH4	C61 H106 O6 N1	948.801	19.075	0.618	0.041	1.402
Group A and Group B						
DG (18:4_18:2)+Li	C39 H64 O5 Li1	619.491	7.509	0.319	0.041	1.390
DG (8:0e_10:4)+NH4	C21 H38 O4 N1	368.280	1.516	0.423	0.020	3.023
DG (20:3e)+NH4	C23 H44 O4 N1	398.326	2.053	0.433	0.011	1.992
DG (18:3e)+NH4	C21 H40 O4 N1	370.295	1.681	0.447	0.004	3.107
DG (20:4e)+NH4	C23 H42 O4 N1	396.311	1.751	0.451	0.030	1.427
MG (18:2)+NH4	C21 H42 O4 N1	372.311	1.922	0.480	0.001	1.370
LPI (20:4)-H	C29 H48 O12 N0 P1	619.289	1.799	0.534	0.045	1.028
PC (18:3_18:2)+HCOO	C45 H79 O10 N1 P1	824.545	8.757	0.556	0.030	2.595
PC (8:1e_10:0)+H	C26 H53 O7 N1 P1	522.355	2.743	0.655	0.031	1.735
PC(10:0e_8:0)+H	C26 H55 O7 N1 P1	524.371	3.685	0.656	0.024	2.539
PC (8:1e_10:1)+HCOO	C27 H51 O9 N1 P1	564.331	2.203	0.664	0.038	3.358
PI (18:0_20:5)-H	C47 H80 O13 N0 P1	883.534	9.881	1.537	0.011	1.932
DG (36:8)+H	C39 H61 O5	609.451	11.387	1.613	0.015	1.886
PE (37:2)+Li	C42 H80 O8 N1 P1 Li1	764.578	11.494	1.685	0.002	6.989
DG (33:2e)+K	C36 H68 O4 K1	603.475	13.297	1.875	0.006	1.180
DG (33:1)+K	C36 H68 O5 K1	619.470	17.617	1.983	0.022	1.215
PS (39:3)+Na	C45 H82 O10 N1 P1 Na1	850.557	10.996	2.271	0.046	4.553

**FIGURE 3:**
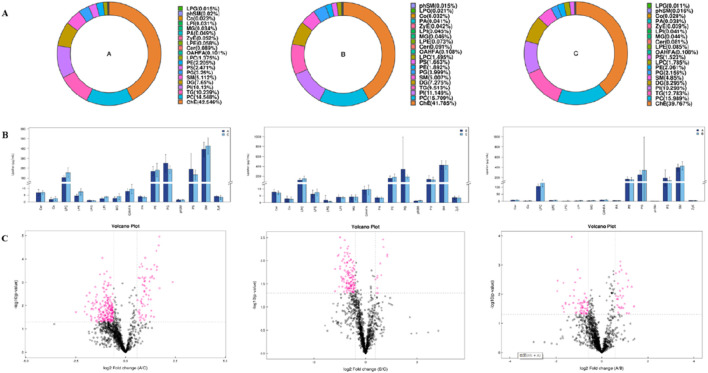
Univariate statistical analysis of lipid metabolites. **(A)** Ring diagram of lipid subclass composition of each group. **(B)** Histogram of differences in lipid subclass content between Groups A and C, Groups B and C, and Groups A and B. **(C)** Volcano plot of differential lipid molecules between Groups A and C, Groups B and C, and Groups A and B.

The main lipid composition and content distribution range of the sample was investigated through lipid composition analysis, of which Figures 3A, B show the results. The differences in lipid subclass content among the left coronary artery occlusion group, right coronary artery occlusion group, and control group were quantitatively analyzed and presented as a histogram. Through comprehensive lipidomic profiling of 1,355 lipid species, we identified 93 differentially expressed lipid molecules (Figure 3C volcano plot). Compared with group C, significantly changed lipid molecules caused by left or right coronary artery occlusion can be clearly observed. The left side of the dotted line represents significantly down-regulated expression, while the right side of the dotted line represents significantly up-regulated expression (P < 0.05).

### 3.3 Multidimensional statistical analysis of lipid metabolites

**FIGURE 4:**
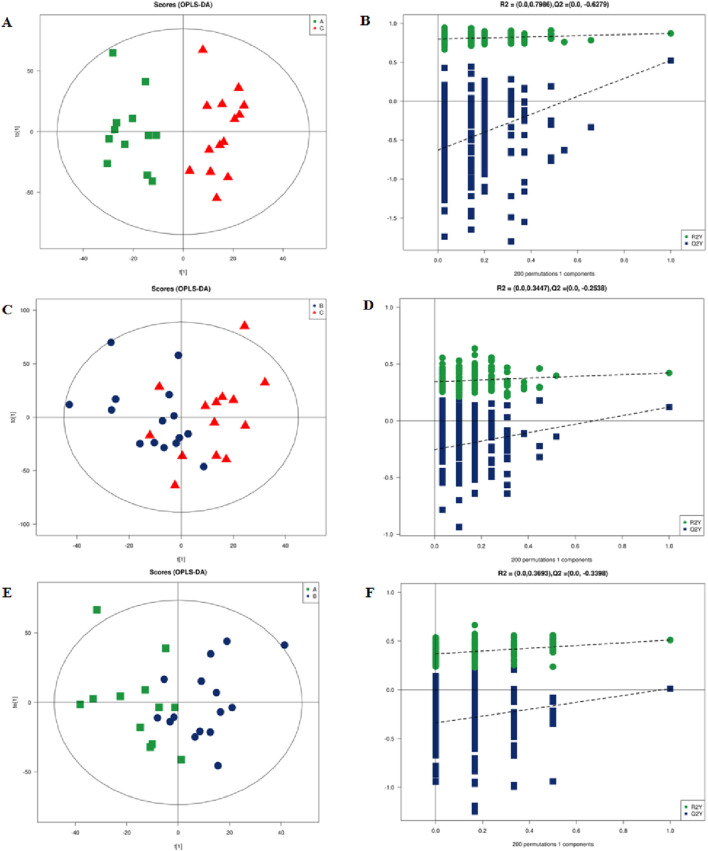
Multidimensional statistical analysis of lipid metabolites. **(A)** OPLS-DA-based plot of acquired MS spectra from Group A (green) and Group C (red). **(B)** Score plot of OPLS-DA model for Groups A and C. **(C)** OPLS-DA-based plot of acquired MS spectra from Group B (blue) and Group C (red). **(D)** Score plot of the OPLS-DA model for Groups B and C. **(E)** OPLS-DA-based plot of acquired MS spectra from Group A (green) and Group B (blue). **(F)** Score plot of OPLS-DA model for Groups A and B: green dots, R2; blue squares, Q2. Green and blue lines represent regression lines for R2 and Q2, respectively. OPLSDA, orthogonal partial least squares discriminant analysis.

We used PCA to examine grouping trends and outliers as an unsupervised data analysis method. In the PCA plot, we observed a clear separation trend in the left coronary artery occlusion group, the right coronary artery occlusion group, and the normal group (Supplementary Figure S1). At the same time, the quality control samples were closely clustered, which also indicated that the instrument was reproducible throughout the analysis period. Pearson’s correlation analysis was performed on the QC samples. The experimental results show that the correlation coefficients between QC samples are all above 0.9, indicating that the experiment has good repeatability. Due to the relatively high dispersion of human plasma samples, we removed three extreme outliers for subsequent analysis. The scatter plots of the OPLS-DA scores based on these signals show an unambiguous difference among the lipid profiles of Groups A, B, and C (Figures 4A,C,E). Figures 4B, D,and F are the permutation test chart of the OPLS-DA model. As the permutation retention gradually decreases, the R2 and Q2 of the random model gradually decrease, indicating that the original model does not have overfitting and that the model is robust.

### 3.4 Cluster analysis

**FIGURE 5:**
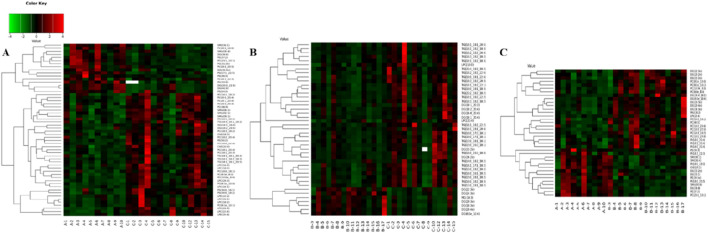
Hierarchical clustering of each sample data set showing differentially expressed lipids. Color is proportional to the intensity of lipid species changes; red color represents up-regulation, and green represents down-regulation. **(A)** Group A compared with Group C. **(B)** Group B compared with Group C. **(C)** Group A compared with Group B.

**FIGURE 6:**
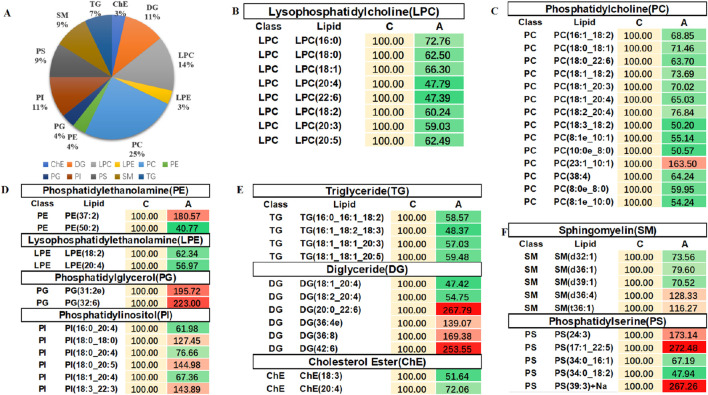
Color scale diagrams of the differential lipid molecules between Groups A and C. **(A)** Analysis of various lipid components. **(B)** Trends of LPC. **(C)** Trends of PC. **(D)** Trends of PE, LPE, PG, and PI. **(E)** Trends of TG, DG, and ChE. **(F)** Trends of SM and PS. LPC, lysophosphatidylcholines; PS, lysophosphatidylcholines; PC, phosphatidylcholine; TG, triglyceride; DG, diglyceride; MG, monoglycerides. PE, phosphatidylethanolamine; des. LPE, lysophosphatidylethanolamine; PG, phosphatidylglycerol; PI, phosphatidylinositol; ChE, cholesterol ester; SM, sphingomyelin.

**FIGURE 7:**
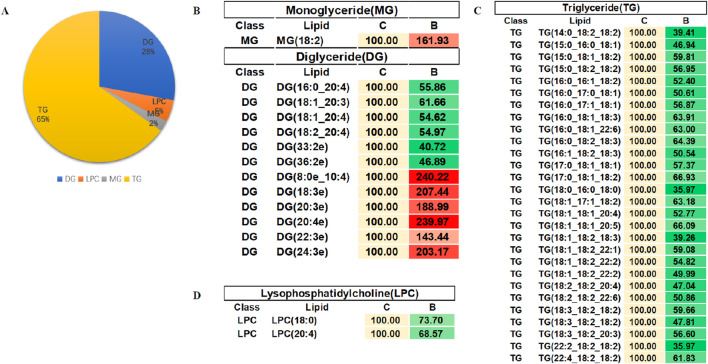
Color scale diagrams of differential lipid molecules between Groups B and C. **(A)** Analysis of various lipid components. **(B)** Trends of MG and DG. **(C)** Trends of TG. **(D)** Trends of LPC. LPC, lysophosphatidylcholines; TG, triglyceride; DG, diglyceride; MG, monoglycerides.

In order to evaluate the rationality of differential lipids and to display relationships between samples and differences in lipid expression patterns across samples comprehensively and intuitively, we used the expression levels of significantly different lipids to perform hierarchical clustering on each group of samples. The same group of samples can appear in the same cluster. Lipids in the same cluster have similar expression patterns and may be in relatively close reaction steps in the metabolic process. From Figure 5A, we found that compared with Group C, Group A had significantly lower detection of LPC class. By comparing Groups B and C (Figure 5B), we found that their differences were mainly concentrated in the glycerol class, including monoglyceride (MG), diglyceride (DG), and triglyceride (TG). Figure 5C shows that the phosphatidylcholine (PC) class was highly expressed in Group B, while the phosphatidylethanolamine (PE) class was highly expressed in Group A. In order to visualize the distribution and expression of differential lipid molecules, we made color scale diagrams of the differential lipid molecules between Groups A and C and Groups B and C respectively in Figures 6 and 7, and the differential lipid molecules between Groups A and B in Supplementary Figure S2.

### 3.5 Assessing the diagnostic value of biomarkers

**FIGURE 8:**
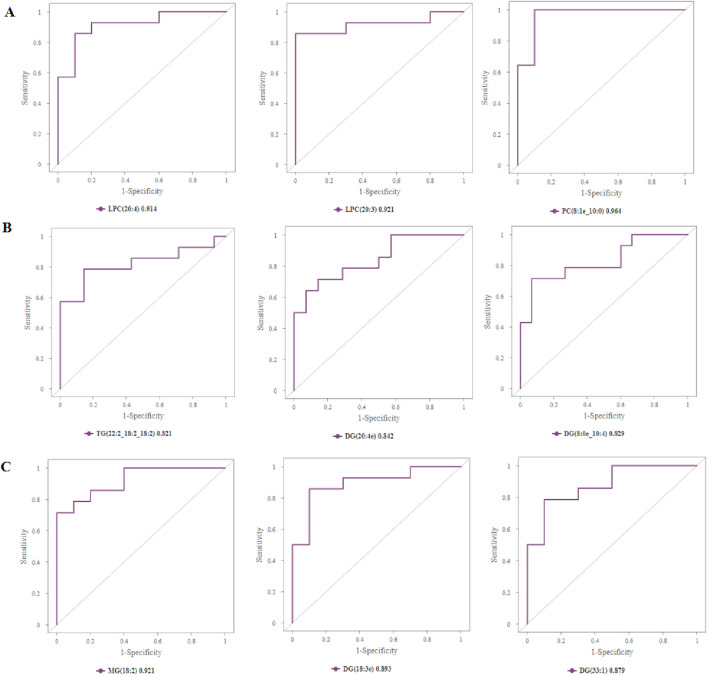
Assessing the diagnostic value of biomarkers. **(A)** Top 3 differential lipid molecules between Groups A and C with an area under the ROC curve >0.9, and the expressions of LPC, PS, and PC in Groups A and C. **(B)** Top 3 differential lipid molecules between Groups B and C with an area under the ROC curve >0.8, and expressions of TG, DG in Groups B and C. **(C)** Top 3 differential lipid molecules between Groups A and B with an area under the ROC curve >0.8, and expressions of MG, DG in Groups A and B. LPC, lysophosphatidylcholines; PS, lysophosphatidylcholines; PC, phosphatidylcholine; TG, triglyceride; DG, diglyceride; MG, monoglycerides.

In order to evaluate the diagnostic value of these differential lipid molecules, we constructed a receiver operating curve (ROC) for the 93 differential lipid molecules with FC > 1.5 or <0.67, P value < 0.01. Among the differential lipid molecules between Groups A and C are 27 lipid molecules with an area under the ROC curve>0.8. We selected the top six lipid molecules with an area under the ROC curve >0.9: LPC (20:4,AUC = 0.914), LPC (20:3, AUC = 0.921), PS (17:1_22:5, AUC = 0.929), PC (8:1e_10:0, AUC = 0.964), PC (8:1e_10:1, AUC = 0.964), PC (23:1_10:1, AUC = 0.943), mainly in the LPC, PS, DG, PC classes (Figure 5A). Among the differential lipid molecules between Groups B and C are nine lipid molecules with ROC curve>0.8, the top six being TG (22:2_18:2_18:2, AUC = 0.821), MG (18:2, AUC = 0.811), DG (24:3e, AUC = 0.816), DG (18:3e, AUC = 0.816), DG (20:4e, AUC = 0.842), DG (8:0e_10:4, AUC = 0.829), mainly in the MG, DG, and TG classes. There are also differences in lipid molecules between patients with left coronary artery occlusion and those with right coronary artery occlusion; these six lipid molecules with an area under the ROC curve>0.8 are MG (18:2, AUC = 0.921), PE (37:2, AUC = 0.886), DG (8:0e_10:4, AUC = 0.85), DG (20:3e, AUC = 0.843), DG (18:3e, AUC = 0.893), DG (33:1, AUC = 0.879), mainly in the MG, PE, and DG classes. These areas under the ROC curve are shown in Figures 8A–C. We also found that some of the lipid molecules also overlapped between Groups A and C and Groups B and C This lipid molecule was LPC (20:4). Since the expression of LPC (20:4) in the two disease groups (A and B) showed a downward trend (P < 0.05, FC < 0.67, VIP > 1), we used it as a biomarker for the diagnosis of myocardial infarction.

### 3.6 Correlation analysis of lipid molecules

**FIGURE 9:**
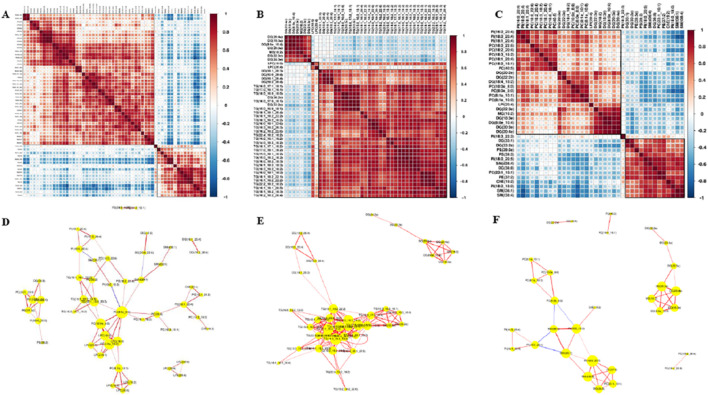
Correlation analysis of lipid molecules. **(A)** Lipid–lipid correlation matrix of differential lipid molecules between Groups A and C. **(B)** Lipid–lipid correlation matrix of differential lipid molecules between Groups B and C. **(C)** Lipid–lipid correlation matrix of differential lipid molecules between Groups A and B, implying the metabolic proximities; red color represents up-correlation, and blue represents down-correlation. **(D)** Network diagram of differential lipid molecules between Groups A and C (p < 0.05, VIP>1). **(E)** Network diagram of differential lipid molecules between Groups B and C. **(F)** Network diagram of differential lipid molecules between Groups A and B.

Since there is no evidence that lipid molecules are directly involved in the GO (Gene Ontology Analysis)/KEGG (Kyoto Encyclopedia of Genes and Genomes Pathway Analysis) pathway analysis, we produced a heat map of the interactions of lipid molecules to analyze the possible network of relationships between these molecules. In this study, we focused on lipid molecules meeting the following criteria: fold change (FC) > 1.5 or <0.67, P-value <0.01 and area under the curve (AUC) > 0.8. 1). Group A vs. C (Figure 9A): correlation analysis of differential lipids revealed a positive association between LPC (20:4) and both LPC (22:6) and LPE (20:3). 2). Group B vs. C (Figure 9B): the interaction network was less complex, with DG (8:0e_10:4) showing positive correlations with DG (20:4e), DG (20:3e), MG (18:2), DG (22:3e), and DG (24:3e). Additionally, LPC (20:4) was positively correlated with LPC (18:0). 3). Group A vs. B (Figure 9C): a significant positive correlation was observed between PE (37:2) and PC(23:1_10:1). These lipid molecules seem to be shining stars in a splendid galaxy, waiting for us to connect the related stars into a constellation with beautiful stories. Network diagram of differential lipid molecules between Groups A and C, Groups B and C, Groups A and B (*p* < 0.05, VIP > 1) were shown in Figures 9D–F.

### 3.7 Bivariate correlation analysis between differential lipids and clinical indicator

**FIGURE 10:**
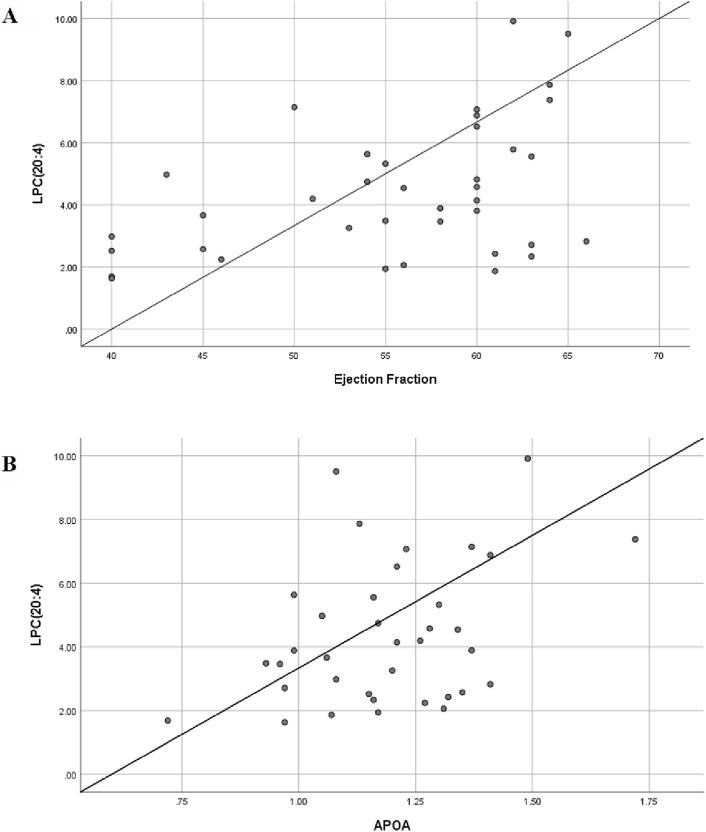
Bivariate correlation analysis between differential lipids and clinical indicator. **(A)** Scatter diagram of correlation between LPC(20:4) and left ventricular ejection fraction; Pearson coefficient is 0.437. **(B)** Scatter diagram of correlation between LPC(20:4) and serum APOA; Pearson coefficient is 0.471.

We were pleasantly surprised to find that LPC (20:4) was positively correlated with patients’ left ventricular ejection fraction (EF value) —the lower the content of LPC (20:4), the lower the left heart ejection fraction and the worse the heart function and clinical prognosis (Figure 10A). The association between APOA, LPC species, and cardiac function was consistent with our clinical findings. APOA—the only significantly altered clinical biomarker in our analysis—demonstrated a strong positive correlation with LPC (20:4) (Figure 10B), suggesting potential interaction between lysophosphatidylcholines (LPCs) and lipoprotein metabolism.

To further explore this relationship, we expanded our correlation analysis to include all detected LPC species and ejection fraction (EF) values. The results revealed significant associations between multiple LPCs and cardiac function parameters (Table 3), reinforcing the potential role of the LPC metabolism in cardiovascular pathophysiology. A moderate degree of correlation was considered when the Pearson correlation coefficient was in the range of 0.4–0.6. We believe that this trend will become more pronounced when the sample size is further expanded in the future.

**TABLE 3:** Correlation analysis between with all obtained LPC and the EF value.

Lipid molecule	*P* value	Pearson correlation coefficient
LPC (16:0)	0.009	0.411
LPC (18:0)	0.006	0.436
LPC (18:2)	0.002	0.477
LPC (20:3)	0.002	0.49
LPC (20:4)	0.005	0.437
LPC (20:5)	0.001	0.512
LPC (22:6)	0.011	0.401

## 4 Discussion

In this study, we performed a comprehensive lipidomic analysis in human artery plasma to investigate lipidomic features from healthy controls and AMI patients with different infarct regions. Subsequent study found significant differences in lipidomics, both between myocardial infarctions with different infarct regions and between myocardial infarction and healthy controls, implying a uniquely altered lipid profile in different locations of AMI. A total of 1,335 molecules were identified, and 93 lipid molecules were differential to distinguish Groups A from C, B from C, and A from B. These results suggest that disordered lipid homeostasis may be related to the onset of AMI.

Atherosclerosis is generally regarded as a chronic inflammatory disease (Lusis, 2000). As we know, arterial blood flow is fast with high oxygen content, and lipid molecules in arterial blood are oxidatively stressed. The occurrence and development of inflammation is closely related to abnormal lipid metabolism and oxidative stress, which are the common pathophysiological changes in the development of atherosclerosis. Therefore, we chose arterial rather than venous plasma for this study.

The anterior descending branch (LAD) runs along the upper ventricular septum (anterior interventricular sulcus) of the right ventricle and the left ventricle, going around the apical notch to the posterior interventricular sulcus. The blood supply area is large, and when it is occluded, the cardiac diastolic and systolic force is significantly reduced or uncoordinated (Hubers et al., 2021). The myocardium supplied by the right coronary artery (RCA) includes the right ventricle. Occlusion of the RCA causes right ventricular infarction. The sharp decrease in right ventricular systolic function can easily lead to a decrease in the left ventricular return blood volume, insufficient filling of the left ventricle, and a decrease in cardiac output (Libby, 2013). Therefore, considering the different function of the left and right coronaries, we grouped patients according to the different branches of the coronary artery.

Through in-depth analysis of clinical data, our study demonstrated that inflammation-related leukocytes, neutrophils, NLR, and hs-CRP in the myocardial infarction patient group were significantly higher than those in the normal group. Numerous studies have shown that atherosclerosis is an inflammatory disease (Sanz and Fayad, 2008). The inflammatory process accompanying AMI is very complex and involves many acute phasing proteins and cell signaling molecules (Knol et al., 2007). An autopsy study of 17 patients who died due to AMI found that CRP was more concentrated in the infarcted cardiac tissue, suggesting that CRP may promote myocardial injury and lead to poor prognosis after AMI (Lagrand et al., 1997). Activated platelets are the core elements of coronary occlusive thrombus in the occurrence of AMI. These activated platelets can bind to WBC and accelerate the occlusive thrombus formation of large coronary arteries and microvessels (Nash, 1994). The larger the infarct size of AMI, the more the myocardial damage, the stronger the inflammatory response, and the higher the WBC count (Grzybowski et al., 2004). Our study also confirmed that hs-CRP is an easily accessible but significant indicator reflecting the inflammatory state of the body. Moreover, lysophosphatidylcholines (LPCs) are highly related to inflammation response, which is also the reason why we choose LPC (20:4) as biomarker metabolites.

This study also found that systolic blood pressure was significantly lower in patients with AMI than in normal controls. In severe AMI patients, the rapid decrease in arterial blood pressure is generally caused by left ventricular dysfunction, which further aggravates myocardial ischemia and necrotic injury (Detollenaere et al., 1993).

Recent studies documenting significant changes in the metabolic profiles of plasma and urinary lipids in cardiovascular disease suggest that these profiles represent potential biomarkers for improving diagnostic efficiency (Lu et al., 2017; Anroedh et al., 2018; Solati and Ravandi, 2019). Lipidomics is a systems-based study of all lipids (Watson, 2006) that has been defined as the full characterization of lipid molecular species and their biological roles (Roberts et al., 2008). A recent cross-sectional study identified 8,9-DiHETrE, a bioactive oxylipin, as being significantly associated with cardiovascular disease (CVD) risk (Caligiuri et al., 2017). This finding adds to growing evidence that implicates oxylipin-mediated pathways in cardiovascular pathophysiology (Caligiuri et al., 2017). There is accumulating evidence that bioactive lipids play important roles in ischemic cardiovascular disease. In the following, we investigate the pathophysiological roles of the differential lipids screened out.

Of the entire lipid subclass level, the LPC class was more significantly reduced in the AMI group (Groups A and B) than that in the normal control group (Group C). ROC curve analysis demonstrated that lysophosphatidylcholine (LPC) species exhibited moderate diagnostic efficacy, with AUC values ranging from 0.914 to 0.921 (Figure 8). These findings suggest that LPC metabolites may serve as potential biomarkers. Since the expression of LPC (20:4) showed a downward trend in both AMI groups (P < 0.05, FC < 0.67, VIP>1) and LPC (20:4) is positively correlated with left ventricular ejection fraction and APOA, we considered LPC (20:4) to be an important biomarker in the pathophysiology of myocardial infarction. LPCs, a type of phospholipids, have a variety of physiological functions and are closely related to metabolic diseases such as diabetes, atherosclerosis, dyslipidemia, and cardiovascular diseases (Schmitz and Ruebsaamen, 2010; Domeij et al., 2013). LPCs exist in small or trace amounts in cell membranes and tissues; they are cytotoxic, causing endothelial cell degeneration, necrosis, and shedding. LPCs can regulate the metabolism of low-density lipoprotein and participate in atherosclerotic lesions caused by sterols. Most recent studies have found that lower LPC plasma levels are associated with unfavorable disease outcomes. Decreased levels of LPC were observed in diabetes (Barber et al., 2012), rheumatoid arthritis (Fuchs et al., 2005), schizophrenia (Wang et al., 2019), polycystic ovary syndrome (Jiang et al., 2019), Alzheimer’s disease (Grösgen et al., 2011), aging (Semba et al., 2019), pulmonary arterial hypertension (Chen et al., 2020), asthma (Baurecht et al., 2013), and liver cirrhosis, where they were associated with increased mortality risk (Krautbauer et al., 2016). These findings strongly corroborate our principal observation that circulating LPC levels are significantly reduced in AMI patients compared to controls. LPC exhibits complex, context-dependent effects on vascular biology. Notably, it demonstrates paradoxical atheroprotective properties through its capacity to mediate cholesterol efflux from lipid-laden macrophages—a mechanism that mirrors HDL’s cardioprotective function (Hou et al., 2007). This finding suggests that LPC may participate in reverse cholesterol transport pathways.

Concurrently, LPC modulates vascular homeostasis by influencing the nitric oxide (NO)–endothelin-1 equilibrium, thereby regulating vascular tone (Gupta et al., 2020). In contrast to these potentially beneficial effects, LPC functions as a potent chemoattractant, recruiting monocytes (Quinn et al., 1988), natural killer cells (Jin et al., 2005), and T lymphocytes (Radu et al., 2004) to inflammatory foci. This chemotactic activity may exacerbate vascular inflammation under certain pathophysiological conditions. Coagulation is a complex process, and LPC was found to not just inhibit platelet aggregation but also to reduce tissue factor activity in monocytes, thereby attenuating coagulation in atherosclerotic lesions (Engelmann et al., 1999). Finally, the ability of LPC to bind C-reactive protein (CRP) and therefore suppress its pro-atherogenic effect on macrophages and delay the progression of atherosclerosis was reported (Chang et al., 2012). Moreover, LPC administration increased coronary blood flow as well as decreasing mean arterial pressure and total vascular resistance in rabbits (Wolf et al., 1991). This could even serve as a promising therapeutic avenue to improve myocardial perfusion and the early correction of myocardial ischemia. Coronary atherosclerosis serves as the pathological foundation of coronary artery disease (CAD). Oxidized low-density lipoprotein (ox-LDL) is one of the primary contributors to atherosclerotic lesions (Steinberg, 2009). Its bioactive component, LPCs, is not only involved in the initiation and progression of atherosclerosis but also closely associated with the early diagnosis and risk assessment of CAD. Galle and Jan were the first to investigate the effects of ox-LDL on vasoconstriction. They discovered that ox-LDL enhances angiotensin II (AngII)-induced vasoconstriction by activating the RhoA signaling pathway. Further studies revealed that LPC fully replicates the causal effects of ox-LDL (Galle et al., 2003). Therefore, we suggest that LPCs should be recognized as important homeostatic mediators involved in all stages of vascular inflammation through their effect on vascular reactivity, endothelial activation and infiltration, the activation of immune cells, and most importantly, on the onset and progression of AMI.

Intergroup comparisons demonstrated a predominant representation of the DG class, particularly in Group B versus C and Group B versus A. DG (8:0e_10:4) levels were significantly higher in Group B relative to the normal controls and Group A (p < 0.05). Diacylglycerol (DAG) is a bioactive lipid that has been shown to reduce fasting serum triglyceride (TG) levels, attenuate postprandial hypertriglyceridemia, decrease glycated hemoglobin (HbA1c) levels in patients with type 2 diabetes mellitus (T2DM), and inhibit ectopic lipid accumulation in both animal models and humans. (Murase et al., 2001). Some have proposed that replacing TG with DAG in the diet is beneficial for the control diabetes and preventing the occurrence of arteriosclerosis and related diseases (Taguchi et al., 2000). Results also show that DAG can effectively improve the lipoprotein status and the ratio of visceral fat and body fat in patients with genetic variants of lipid transporters (Yanagisawa et al., 2003). By increasing the β-oxidation of fatty acids (Murata et al., 1997), affecting the expression of genes related to lipid metabolism, and over-regulating the co-activators of the PPAR family (Murase et al., 2002), DAG can control body weight and reduce visceral fat content. Current studies show that the lipid metabolism in gut microbes may be associated with atherosclerosis and that elevated levels of phosphatidylcholine (PC) are positively correlated with the risk of cardiovascular disease. This phenomenon may be associated with trimethylamine N-oxide (TMAO), a gut microbiota-dependent metabolite derived from phosphatidylcholine metabolism. High levels of TMAO in the body can affect the cholesterol metabolism, promote the formation of atherosclerotic plaques, and aggravate lipid metabolism disorders (Wang et al., 2011). Phosphatidylcholine is the main food source for the formation of TMAO, and it serves as the primary dietary precursor for trimethylamine N-oxide (TMAO) formation; TMAO has been identified as a key mediator in phosphatidylcholine-induced cardiovascular disease pathogenesis (Koeth et al., 2013; Tang et al., 2013).

## 5 Conclusion

This study demonstrated that lipid metabolism in patients with AMI in different infarct regions was significantly changed compared with normal controls. After ROC screening, those with higher diagnostic values belonged to the following categories: LPC, PS, PC, PE, DG, and MG. We finally focused on the LPC (20:4) molecule as a biomarker metabolite. These lipidomics findings provide valuable insights for the further investigation of potential biomarkers and pathological mechanisms underlying AMI.

Given the limited sample size in this study, larger-scale validation studies are required to confirm the functional roles of differentially expressed lipid species in AMI patients across various stages of coronary artery disease. The standardization of mass spectrometry-based quantification and analytical methodologies is essential to enable high-throughput cohort analyses. Such standardization will facilitate a more precise understanding of the pathophysiological contributions of individual lipid molecules in AMI. In subsequent research, we will establish a mouse myocardial infarction model by ligating the left anterior descending coronary artery. We will specifically assess changes in LPC (20:4) content and its effects on inflammatory and immune signaling within cardiomyocytes following ischemia/hypoxia. In addition, we will verify the function of LPC (20:4) in myocardial ischemia.

## Data Availability

The datasets presented in this article are not readily available; the data that support the findings of this study are available from the corresponding author upon reasonable request. Requests to access the datasets should be directed to Xing Li, lixing880216@126.com.

## Supplementary material

SUPPLEMENTARY FIGURE S1 PCA of Groups A, B, C, and QC. Two outliers (black arrow) in Group B and one (blue arrow) in Group C were excluded, and 41 samples were finally included for analysis. PCA, principal component analysis; QC, quality control.

SUPPLEMENTARY FIGURE S2 Color scale diagrams of the differential lipid molecules between Groups A and B. **(A)** Analysis of various lipid components. **(B)** Trends of DG. **(C)** Trends of PC. **(D)** Trends of ChE, PE, PS, and SM. **(E)** Trends of LPI. **(F)** Trends of PI. PS, lysophosphatidylcholines; PC, phosphatidylcholine; DG, diglyceride; PE, phosphatidylethanolamine; PI, phosphatidylinositol; ChE, cholesterol ester; SM, sphingomyelin; LPI, lysophosphatidylinositol.
