# Supplementary material for: Identification of plasma lipid metabolism and potential biomarkers in patients with different coronary occlusive acute myocardial infarction
Source: Front Cell Dev Biol. 2025 Aug 14;13:1575431. doi: 10.3389/fcell.2025.1575431 (PMC12391077; doi:10.3389/fcell.2025.1575431)
Supplement: Supplementary file 1 [file DataSheet1.docx]

**Supplemental Figure1**


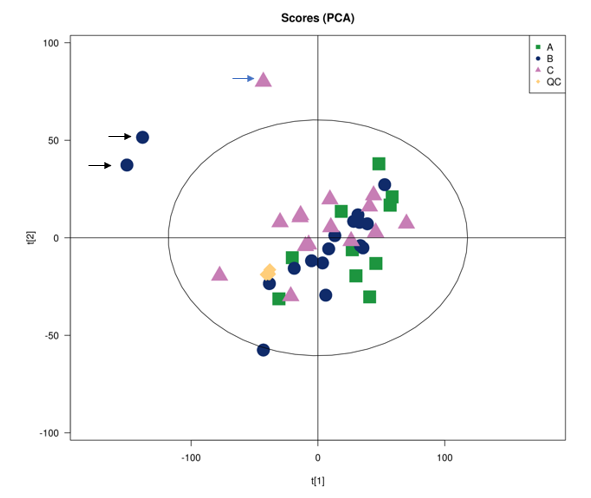


**Supplementary Figure S1**. Principal Component Analysis (PCA) of Group A, Group B, Group C and quality control (QC). Two outliers (black arrow) in Group B and one outlier (blue arrow) in Group C were excluded, and 41 samples were finally included for analysis.


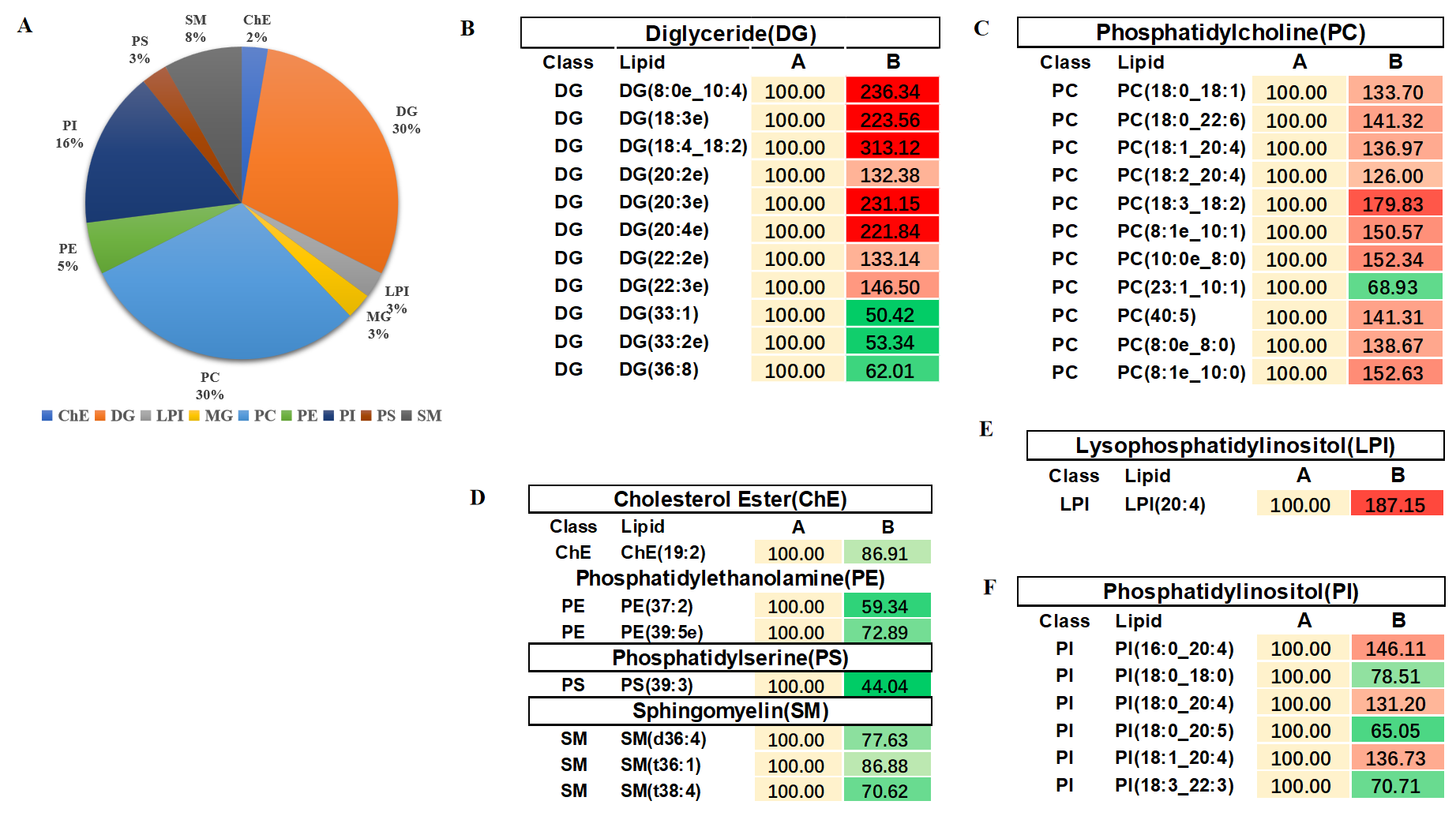


**Supplementary Figure S2.** The color scale diagrams of the differential lipid molecules between Group A and B. (A)Analysis of various lipid components. (B)Trends of DG. (C)Trends of PC. (D)Trends of ChE, PE, PS and SM. (E)Trends of LPI. (F)Trends of PI.
